# *MET* amplification as a potential therapeutic target in gastric cancer

**DOI:** 10.18632/oncotarget.718

**Published:** 2012-11-17

**Authors:** Hisato Kawakami, Isamu Okamoto, Tokuzo Arao, Wataru Okamoto, Kazuko Matsumoto, Hirokazu Taniguchi, Kiyoko Kuwata, Haruka Yamaguchi, Kazuto Nishio, Kazuhiko Nakagawa, Yasuhide Yamada

**Affiliations:** ^1^ Department of Medical Oncology, Kinki University Faculty of Medicine, 377-2 Ohno-higashi, Osaka-Sayama, Osaka, Japan; ^2^ Department of Genome Biology, Kinki University Faculty of Medicine, 377-2 Ohno-higashi, Osaka-Sayama, Osaka, Japan; ^3^ Gastrointestinal Medical Oncology Division, National Cancer Center Hospital, Tokyo, Japan; ^4^ Department of Pathology, National Cancer Center Hospital, Tokyo, Japan

**Keywords:** MET, gastric cancer, gene amplification, FISH, PCR

## Abstract

Our aim was to investigate both the prevalence of MET amplification in gastric cancer as well as the potential of this genetic alteration to serve as a therapeutic target in gastric cancer. MET amplification was assessed by initial screening with a PCR-based copy number assay followed by confirmatory FISH analysis in formalin-fixed, paraffin-embedded specimens of gastric cancer obtained at surgery. The effects of MET tyrosine kinase inhibitors (MET-TKIs) in gastric cancer cells with or without MET amplification were also examined. The median MET copy number in 266 cases of gastric cancer was 1.7, with a range of 0.41 to 21.3. We performed FISH analysis for the 15 cases with the highest MET copy numbers. MET amplification was confirmed in the four assessable cases with a MET copy number of at least 4, whereas MET amplification was not detected in those with a gene copy number of <4. The prevalence of MET amplification was thus 1.5% (4 out of 266 cases). Inhibition of MET by MET-TKIs resulted in the induction of apoptosis accompanied by attenuation of downstream MET signaling in gastric cancer cell lines with MET amplification but not in those without this genetic change. MET amplification identifies a small but clinically important subgroup of gastric cancer patients who are likely to respond to MET-TKIs. Furthermore, screening with a PCR-based copy number assay is an efficient way to reduce the number of patients requiring confirmation of MET amplification by FISH analysis.

## INTRODUCTION

Gastric cancer is the third most common cause of death from malignant disease in men (fifth in women) worldwide [1]. The prognosis for patients with unresectable advanced or recurrent gastric cancer remains poor, with a median survival time of less than 1 year in individuals receiving conventional therapy [2-8]. The combination of trastuzumab, an antibody targeted to HER2, with chemotherapy has yielded a survival benefit for patients with HER2-positive gastric or gastro-esophageal junction cancer [7], with HER2-positive tumors accounting for 7 to 17% of all gastric cancers [9-11]. Further research is thus warranted to identify new therapeutic targets for gastric cancer patients.

The *MET* proto-oncogene encodes the receptor tyrosine kinase c-MET. The binding of its ligand, hepatocyte growth factor, to MET results in tyrosine phosphorylation of the receptor and activation of downstream signaling molecules. Oncogenic activation of *MET* suppresses apoptosis and promotes cell survival, proliferation, migration, and differentiation as well as gene transcription and angiogenesis [12]. In gastric cancer, such activation of *MET* has been attributed to gene amplification [13-15]. However, the prevalence of *MET* amplification has varied among studies [13-21], possibly as a result of differences in the methods applied. This uncertainty led us to determine the prevalence of *MET* amplification in 266 formalin-fixed, paraffin-embedded (FFPE) specimens of gastric cancer obtained during surgery. To ensure the efficient detection of *MET* amplification, we adopted a sequential approach involving PCR-based determination of gene copy number followed by confirmatory FISH analysis. Moreover, to assess the potential of *MET* amplification as a therapeutic target in gastric cancer, we investigated its impact on cell survival and signal transduction.

## RESULTS

### MET amplification in gastric cancer cell lines

**Figure 1:**
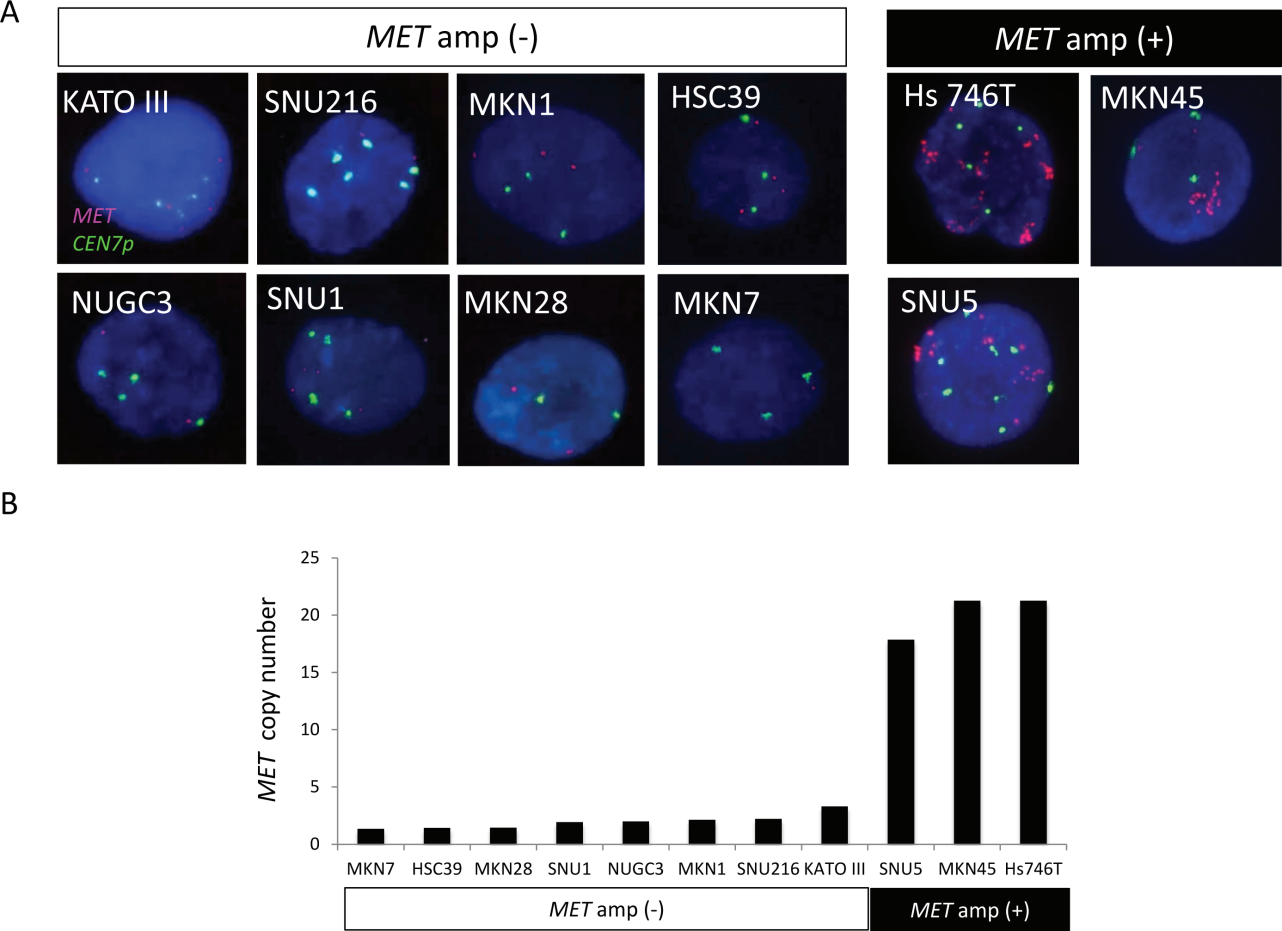
Amplification of *MET* in gastric cancer cell lines *A*, FISH analysis of cell lines positive or negative for *MET* amplification (amp). Each image shows a single cancer cell, with green and red signals corresponding to CEN7p and the *MET* locus, respectively. *B*, Evaluation of *MET* copy number in gastric cancer cell lines with a PCR-based assay.

We first applied FISH (Figure 1A) and a real-time PCR–based method (Figure 1B) to examine *MET* copy number in gastric cancer cell lines whose *MET* amplification status was previously determined [22]. In gastric cancer cell lines negative for *MET* amplification, including KATO III, SNU1, SNU216, MKN1, MKN7, HSC39, MKN28, and NUGC3, the copy number of *MET* as determined by the PCR-based assay ranged between 1.3 and 3.3. In contrast, cell lines positive for *MET* amplification, including Hs746T, MKN45, and SNU5, showed *MET* copy numbers of 21.3, 21.3, and 17.9, respectively. The PCR-based assay thus revealed a high copy number for *MET* only in gastric cancer cell lines previously shown to be positive for *MET* amplification by FISH.

### MET amplification in gastric cancer specimens

To determine the prevalence of *MET* amplification in advanced gastric cancer, we examined 266 FFPE specimens of surgically resected primary gastric tumors. Most of the patients were male (68.8%) and had undifferentiated-type gastric cancer (62.8%), including mucinous adenocarcinoma, signet ring cell adenocarcinoma, and poorly differentiated adenocarcinoma (Table 1). The median age was 63 years, with a range of 31 to 91 years.

**Table 1:** Characteristics of the 266 study patients

Characteristic	n
**Sex** Male	183 (68.8%)
Female	83 (31.2%)
**Pathological stage**	
I	25 (9.4%)
II	31 (11.7%)
III	77 (28.9%)
IV	133 (50.0%)
**Histology**	
Differentiated type	99 (37.2%)
Undifferentiated type	167 (62.8%)

The patients had a median age of 63 years (range, 31 to 91 years).

**Figure 2:**
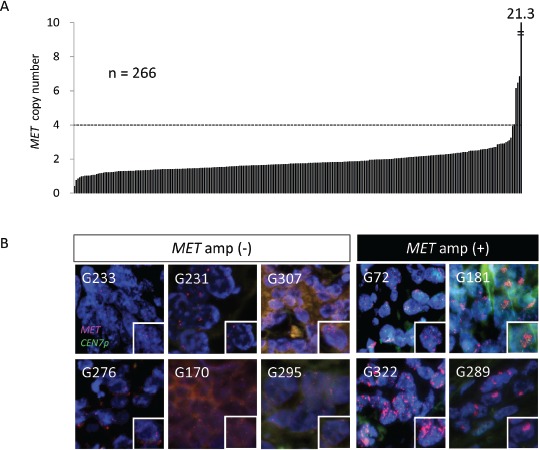
Amplification of *MET* in surgical specimens of gastric cancer *A*, *MET* copy number determined with a PCR-based assay for 266 FFPE surgical specimens of gastric cancer. A *MET* copy number of >4 was observed in five cases. *B*, FISH analysis of gastric cancer specimens among the 15 samples with the highest *MET* copy numbers as determined with the PCR-based assay. Green and red signals correspond to CEN7p and the *MET* locus, respectively. Higher magnification images of individual cancer cells are shown in the insets. The specimens are grouped into those determined to be positive of negative for *MET* amplification by FISH.

The PCR-based assay revealed that the median *MET* copy number for the 266 cases was 1.7, with a range of 0.41 to 21.3 copies (Figure 2A). Given that gastric cancer cell lines with *MET* amplification have been found to have a high copy number for *MET* [23], we arranged all cases in the order of *MET* copy number and performed FISH analysis for the 15 cases with the highest copy numbers (Table 2). *MET* amplification was detected by FISH in four of these cases (G72, G289, G322, and G181), which had a *MET* copy number of at least 4, whereas six cases (G276, G233, G295, G170, G307, and G231) with a copy number of less than 4 did not exhibit *MET* amplification (Figure 2B, Table 2). The remaining five cases (G331, G223, G217, G118, and G42) were not assessable by FISH analysis because of a lack of hybridization signals.

**Table 2:** Characteristics of the 15 patients with the highest MET copy numbers

Case no.	MET copy number	MET/CEN7p	Sex	Age (years)	Histology	Stage	OS (days)
G72	21.3	5.9 (+)	M	66	U	IV	157
G289	6.84	5.2 (+)	F	48	U	IV	483
G322	6.45	7.2 (+)	F	70	U	IV	84
G331	6.14	ND	M	76	U	II	>2764
G181	4.02	6.6 (+)	F	52	U	IIIb	>1977
G223	3.92	ND	M	62	U	IIIa	>3340
G276	3.23	1.2 (−)	M	70	U	Ib	1089
G233	3.09	1.2 (−)	F	63	U	IIIb	>4732
G217	3.02	ND	F	69	U	IIIa	>3827
G118	2.97	ND	M	66	U	IIIa	2650
G295	2.89	2.0 (−)	M	74	U	IV	539
G170	2.88	1.7 (−)	M	53	D	Ia	>2088
G42	2.87	ND	M	64	D	II	1907
G307	2.85	1.3 (−)	M	60	U	IV	824
G231	2.83	1.9 (−)	M	67	D	IIIa	>2921

Abbreviations: ND, signals not detected; U, undifferentiated type; D, differentiated type. (+) or (−) denote positive or negative for MET amplification on the basis of the MET/CEN7p ratio; > for OS indicates the patient was still alive.

We thus identified four out of 266 gastric cancer patients (1.5%) as having *MET* amplification. The clinical features of patients with or without *MET* amplification are shown in Tables 2 and 3. All four patients with *MET* amplification had undifferentiated-type gastric cancer. We further examined the prognostic impact of *MET* amplification for all 266 patients but found that OS after surgery did not differ significantly between those with or without *MET* amplification (log-rank test, *P* = 0.3).

**Table 3:** Clinical and pathological characteristics of gastric cancer patients classified according to MET amplification status

Characteristic	MET amplification(+) (n = 4)	MET amplification(−) (n = 262)	P
Median age (range), years	59 (48–70)	63 (31–91)	0.976
Sex, n			
Male	1 (25.0%)	182 (69. 5%)	0.091
Female	3 (75.0%)	80 (30. 5%)	
Pathological stage, n			
I	0	25 (9.5%)	0.582^a^
II	0	31 (11.8%)	
III	1 (25.0%)	76 (29.0%)	
IV	3 (75.0%)	130 (49.6%)	
Histology, n			
Differentiated type	0	99 (37.8%)	0.300^b^
Undifferentiated type	4 (100%)	163 (62.2%)	

a Comparison between stages I + II and III + IV.

b Comparison between intestinal-type and diffuse-type gastric cancer. P values were calculated with Student's two-tailed t test for age and the chi-square test for the other variables.

### MET amplification is associated with increased sensitivity to MET-TKIs in gastric cancer cell lines

**Figure 3:**
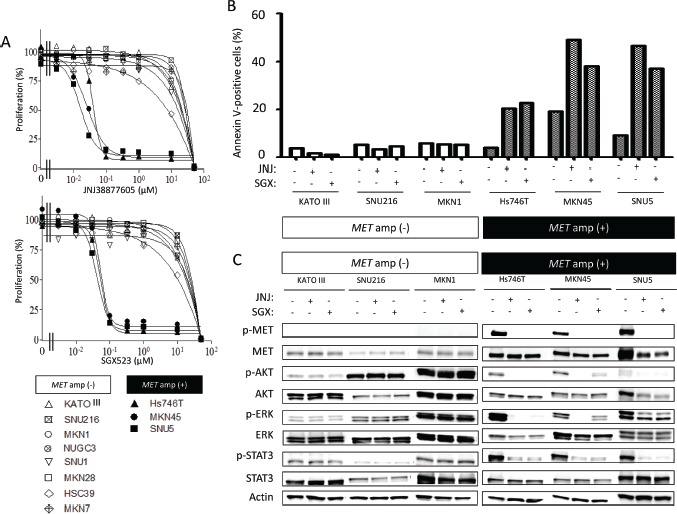
Effects of MET-TKIs in human gastric cancer cell lines classified according to *MET* amplification status *A*, Effects of JNJ38877605 and SGX523 on cell growth as determined with the MTT assay. Data are means of triplicates from representative experiments. *B*, Cells were incubated in the absence or presence of 0.10 μM JNJ38877605 or 0.10 μM SGX523 for 48 h, after which the number of apoptotic cells was determined by staining with annexin V followed by flow cytometry. *C*, Cells were incubated in the absence or presence of 0.10 μM JNJ38877605 or 0.10 μM SGX523 for 48 h, after which cell lysates were prepared and subjected to immunoblot analysis with antibodies to phosphorylated (p) or total forms of MET, AKT, ERK, or STAT3 or with those to β-actin (loading control).

To investigate the biological impact of *MET* amplification in gastric cancer, we first examined the effects of two highly selective MET receptor tyrosine kinase inhibitors (MET-TKIs), JNJ38877605 and SGX523, on the growth of gastric cancer cell lines positive or negative for *MET* amplification. The IC_50_ values of JNJ3887605 and SGX523 for inhibition of cell growth were 0.02 to 0.05 μM and 0.06 to 0.07 μM, respectively, for cells positive for *MET* amplification, whereas they were >10 μM for *MET* amplification–negative cells (Figure 3A). An annexin V binding assay revealed that both MET-TKIs induced a substantial level of apoptosis in *MET* amplification–positive cells but were largely without effect in cell lines without *MET* amplification (Figure 3B). Immunoblot analysis showed that the MET-TKIs inhibited the phosphorylation of MET, AKT, ERK, and STAT3 in gastric cancer cells with *MET* amplification, whereas they had no effect on signaling events in those negative for *MET* amplification (Figure 3C). These findings thus indicated that gastric cancer cells with *MET* amplification are predominantly dependent on MET signaling for their growth and survival and are therefore rendered hypersensitive to MET-TKIs.

## DISCUSSION

Activation of MET signaling promotes tumor cell growth, survival, migration, and invasion as well as tumor angiogenesis [24]. In gastric cancer, gain-of-function mutations of *MET* are exceedingly rare [25-27], with MET activation having been attributed mostly to gene amplification [13-15]. Previous studies based on FISH analysis have detected *MET* amplification in up to 4% of patients with gastric cancer [14, 16, 20]. On the other hand, an increase in *MET* copy number was found in 10 to 20% of gastric cancer patients by Southern blot analysis [17-19] or with a PCR-based assay [28, 29]. An increase in gene copy number in malignant tumors can result from at least two genetic mechanisms, gene amplification and polysomy. Gene amplification refers to a copy number gain for a specific gene (or group of genes) on a given chromosome arm without a change in copy number for genes located in other regions of the chromosome [30], whereas polysomy gives rise to a copy number gain for a given gene as a result of the presence of extra copies of the entire chromosome. Southern blot analysis and PCR-based copy number assays recognize a gain in gene copy number regardless of the underlying cause and are thus unable to distinguish gene amplification from polysomy, a limitation that is sometimes overlooked, with consequences for determination of the true prevalence of *MET* amplification in gastric cancer.

FISH analysis is a semiquantitative method that can be performed with two probes for determination of the number of signals for a target gene and for the centromeric portion of the corresponding chromosome. Given that the number of centromeric signals directly reflects the copy number of the chromosome, FISH analysis yields the copy number gain for the target gene from the ratio of the copy number of the gene to that of the chromosome. FISH is thus the gold standard for detection of gene amplification. However, the identification and counting of FISH signals are hampered by many factors including cutting artifacts, nuclear overlap, and heterogeneity of tumor specimens. Moreover, FISH is expensive and time-consuming, and it requires technical expertise [31]. The efficient determination of gene amplification in a large number of tumor specimens would thus benefit from the availability of a high-throughput screening assay. In this regard, PCR-based assays for determination of gene copy number are simple to perform and rapidly yield quantitative and reproducible results. Given that, among tumors showing a gain in gene copy number, those confirmed to be positive for gene amplification show the greatest increases in gene copy number [21, 23, 32, 33], we investigated the potential of a PCR-based assay for screening in order to select cases of gastric cancer for confirmation of *MET* amplification by FISH. We thus performed PCR-based screening for *MET* copy number in 266 surgically resected specimens of gastric cancer and then applied FISH analysis to the 15 cases showing the highest gene copy numbers. *MET* amplification was confirmed by FISH in four cases among the five with a *MET* copy number of at least 4; the remaining case (G331) was not assessable by FISH because of a lack of hybridization signals. *MET* amplification was not detected in the cases with a gene copy number of <4. We therefore identified *MET* amplification at a frequency of 1.5% (4 out of 266 cases), consistent with values determined by FISH analysis in recent studies of gastric cancer [16, 20]. Our results thus suggest that screening for *MET* amplification with a PCR-based assay is an efficient means with which to reduce the number of specimens requiring evaluation by FISH analysis. As mentioned above, one specimen (G331) in the present study showed a high *MET* copy number (6.14 copies) but could not be confirmed positive for *MET* amplification because of the lack of a FISH result. The issue of how to identify *MET* amplification status in such cases remains to be resolved.

We examined the biological impact of *MET* amplification in gastric cancer cells by comparing the effects of the MET-TKIs JNJ38877605 and SGX523 between gastric cancer cell lines positive for *MET* amplification and those negative for this genetic alteration. In gastric cancer cells with *MET* amplification, the MET-TKIs markedly inhibited AKT, ERK, and STAT3 signaling and triggered apoptosis, whereas such effects were not evident in cells without *MET* amplification. To investigate whether attenuation of MET signaling by the MET-TKIs is related to the induction of apoptosis, we transfected gastric cancer cell lines with an siRNA specific for MET mRNA. Such transfection inhibited MET signal transduction as well as induced apoptosis in gastric cancer cell lines with *MET* amplification but not in those without it (data not shown). Our observations thus indicate that gastric cancer cell lines positive for *MET* amplification depend predominantly on constitutive activation of the encoded growth factor receptor for their survival and thus show high sensitivity to cell killing by MET-TKIs. Targeting of MET signaling by MET-TKIs is therefore a potentially valuable therapeutic approach for patients with *MET* amplification–positive gastric cancer. Indeed, the MET-TKI crizotinib (PF-02341066) was recently found to induce a radiographic response (partial response) and rapid clinical improvement in patients with advanced gastric cancer who were found to be positive for *MET* amplification by FISH [16]. Further investigation of the efficacy of MET-TKIs in patients with advanced gastric cancer positive for *MET* amplification is thus warranted.

Given the potential of MET-targeted therapy for gastric cancer with *MET* amplification, it is important to determine the prevalence of such gene amplification in patients with unresectable advanced gastric cancer, most of whom are currently treated with systemic chemotherapy. Our present study was limited to gastric cancer patients who underwent gastrectomy, and so further studies will be needed for patients with unresectable advanced tumors. Given the apparent low prevalence of *MET* amplification in gastric cancer, implementation of a sequential approach including screening with a PCR-based copy number assay followed by confirmatory FISH analysis should facilitate the identification of *MET* amplification in a large cohort of patients with unresectable advanced gastric cancer.

## MATERIALS AND METHODS

### Cell culture

The human gastric cancer cell lines SNU1, SNU5 and Hs746T were obtained from American Type Culture Collection (Manassas, VA); MKN1, MKN7, MKN45, and NUGC3 were from the Health Science Research Resources Bank (Japan Health Sciences Foundation, Tokyo, Japan); KATO III, MKN28, and HSC39 were from Immuno-Biological Laboratories (Gunma, Japan); and SNU216 was from the Korean Cell Line Bank (Seoul National University, Seoul, Korea). All of the cell lines were maintained under a humidified atmosphere of 5% CO_2_ at 37°C in RPMI 1640 medium (Sigma, St. Louis, MO) supplemented with 10% heat-inactivated FBS (Gibco BRL, Grand Island, NY), penicillin, and streptomycin.

### Patients

A total of 267 patients with histologically confirmed gastric cancer who had undergone surgery at the National Cancer Center Hospital (Tokyo, Japan) between 1996 and 2006 were included in the study. All the patients had an Eastern Cooperative Oncology Group performance status of 0 to 2. One patient was subsequently excluded as a result of an insufficient quantity of DNA extracted from the corresponding tissue specimen. The specimens from the remaining 266 patients were thus analyzed. The present study was approved by the Institutional Review Board of the National Cancer Center Hospital, and informed consent was obtained from all subjects.

### Isolation of genomic DNA

Macrodissection of the surgical specimens preserved as FFPE tissue was performed after removal of paraffin in order to select a region of cancer tissue. Genomic DNA was extracted from the cancer tissue with the use of a QIAamp DNA Micro Kit (Qiagen, Hilden, Germany). The DNA concentration of the extracts was determined with a NanoDrop 2000 spectrophotometer (Thermo Scientific, Waltham, MA).

### PCR-based determination of MET copy number

The copy number of *MET* was determined with the use of a TaqMan Copy Number Assay [32] and the Hs05005660_cn (intron 16) primer (Applied Biosystems, Foster City, CA). The *TERT* locus was used as the internal reference, and DNA from noncancerous FFPE tissue was used as a normal control. Real-time PCR was performed in a total volume of 20 μL per well containing 10 μL of TaqMan genotyping master mix, 20 ng of genomic DNA, and each primer. The amplification protocol included an initial incubation at 95°C for 10 min followed by 40 cycles of 95°C for 15 s and 60°C for 1 min. The resulting products were detected with the use of ABI Prism 7900HT Sequence Detection System (Applied Biosystems). Data were analyzed with SDS 2.2 software and Copy Caller software (Applied Biosystems).

### FISH

*MET* copy number per cell was determined by FISH with the use of the c-met / CEN7p Dual Color FISH Probe (GSP Laboratory, Kawasaki, Japan) [22], where CEN7p is the centromeric region of chromosome 7p. The signals were detected by fluorescence microscopy and were evaluated by independent observers (H.K. and I.O.). After screening all entire sections, images of tumor cells were captured and recorded and the signals for 60 random nuclei were counted for an area where individual cells were recognized in at least 10 representative images. Nuclei with a disrupted boundary were excluded from the analysis. Gene amplification was strictly defined by a mean *MET*/CEN7p copy number ratio of >2.2, corresponding to a previous definition of *MET* amplification [16]. The presence of polysomy or an equivocal *MET*/CEN7p ratio (1.8 to 2.2) were thus scored as negative for amplification.

### Immunoblot analysis

Immunoblot analysis was performed as described previously [22]. Rabbit polyclonal antibodies to phosphorylated human MET (pY1234/pY1235), to total AKT, to phosphorylated AKT, to phosphorylated extracellular signal–regulated kinase (ERK), to phosphorylated or total forms of STAT3 were obtained from Cell Signaling Technology (Danvers, MA); those to total ERK were from Santa Cruz Biotechnology (Santa Cruz, CA); those to total MET were from Zymed/Invitrogen (Carlsbad, CA); and those to β-actin were from Sigma. All antibodies were used at a 1:1000 dilution, with the exception of those to β-actin (1:200).

### Cell growth inhibition assay

Cells were transferred to 96-well flat-bottomed plates and cultured for 24 h before exposure to various concentrations of JNJ38877605 (Janssen Pharmaceutica NV, Beerse, Belgium) or SGX523 (SGX Pharmaceuticals, San Diego, CA) for 72 h. Tetra Color One (5 mmol/L tetrazolium monosodium salt and 0.2 mmol/L 1-methoxy-5-methyl phenazinium methylsulfate; Seikagaku Kogyo, Tokyo, Japan) was then added to each well, and the cells were incubated for 3 h at 37°C before measurement of absorbance at 490 nm with a Multiskan Spectrum instrument (Thermo Labsystems, Boston, MA). Absorbance values were expressed as a percentage of that for nontreated cells, and the IC_50_ values of JNJ38877605 and SGX523 for inhibition of cell growth were determined.

### Annexin V binding assay

The binding of annexin V to cells was measured with the use of an Annexin-V-FLUOS Staining Kit (Roche, Basel, Switzerland). Cells were harvested by exposure to trypsin-EDTA, washed with PBS, and centrifuged at 200 × *g* for 5 min. The cell pellets were resuspended in 100 μL of Annexin-V-FLUOS labeling solution, incubated for 10 to 15 min at 15° to 25°C, and then analyzed for fluorescence with a flow cytometer (FACSCalibur) and Cell Quest software (Becton Dickinson, Franklin Lakes, NJ).

### Statistical analysis

Overall survival (OS) curves were estimated with the Kaplan-Meier method and compared with the log-rank test. Other statistical analysis was performed with Student's two-tailed *t* test or the chi-square test. A *P* value of <0.05 was considered statistically significant.

## References

[1] Jemal A, Bray F, Center MM, Ferlay J, Ward E, Forman D (2011). Global cancer statistics. CA Cancer J Clin.

[2] Van Cutsem E, Moiseyenko VM, Tjulandin S, Majlis A, Constenla M, Boni C, Rodrigues A, Fodor M, Chao Y, Voznyi E, Risse ML, Ajani JA (2006). Phase III study of docetaxel and cisplatin plus fluorouracil compared with cisplatin and fluorouracil as first-line therapy for advanced gastric cancer: a report of the V325 Study Group. J Clin Oncol.

[3] Cunningham D, Starling N, Rao S, Iveson T, Nicolson M, Coxon F, Middleton G, Daniel F, Oates J, Norman AR (2008). Capecitabine and oxaliplatin for advanced esophagogastric cancer. N Engl J Med.

[4] Koizumi W, Narahara H, Hara T, Takagane A, Akiya T, Takagi M (2008). S-1 plus cisplatin versus S-1 alone for first-line treatment of advanced gastric cancer (SPIRITS trial): a phase III trial. Lancet Oncol.

[5] Kang YK, Kang WK, Shin DB, Chen J, Xiong J, Wang J, Lichinitser M, Guan Z, Khasanov R, Zheng L, Philco-Salas M, Suarez T, Santamaria J, Forster G, McCloud PI (2009). Capecitabine/cisplatin versus 5-fluorouracil/cisplatin as first-line therapy in patients with advanced gastric cancer: a randomised phase III noninferiority trial. Ann Oncol.

[6] Ajani JA, Rodriguez W, Bodoky G, Moiseyenko V, Lichinitser M, Gorbunova V, Vynnychenko I, Garin A, Lang I, Falcon S (2010). Multicenter phase III comparison of cisplatin/S-1 with cisplatin/infusional fluorouracil in advanced gastric or gastroesophageal adenocarcinoma study: the FLAGS trial. J Clin Oncol.

[7] Bang YJ, Van Cutsem E, Feyereislova A, Chung HC, Shen L, Sawaki A (2010). Trastuzumab in combination with chemotherapy versus chemotherapy alone for treatment of HER2-positive advanced gastric or gastro-oesophageal junction cancer (ToGA): a phase 3, open-label, randomised controlled trial. Lancet.

[8] Ohtsu A, Shah MA, Van Cutsem E, Rha SY, Sawaki A, Park SR, Lim HY, Yamada Y, Wu J, Langer B, Starnawski M, Kang YK (2011). Bevacizumab in Combination With Chemotherapy As First-Line Therapy in Advanced Gastric Cancer: A Randomized, Double-Blind, Placebo-Controlled Phase III Study. J Clin Oncol.

[9] Hofmann M, Stoss O, Shi D, Buttner R, van de Vijver M, Kim W, Ochiai A, Ruschoff J, Henkel T (2008). Assessment of a HER2 scoring system for gastric cancer: results from a validation study. Histopathology.

[10] Tanner M, Hollmen M, Junttila TT, Kapanen AI, Tommola S, Soini Y, Helin H, Salo J, Joensuu H, Sihvo E, Elenius K, Isola J (2005). Amplification of HER-2 in gastric carcinoma: association with Topoisomerase IIalpha gene amplification, intestinal type, poor prognosis and sensitivity to trastuzumab. Ann Oncol.

[11] Gravalos C, Jimeno A (2008). HER2 in gastric cancer: a new prognostic factor and a novel therapeutic target. Ann Oncol.

[12] Birchmeier C, Birchmeier W, Gherardi E, Vande Woude GF (2003). Met, metastasis, motility and more. Nat Rev Mol Cell Biol.

[13] Nakajima M, Sawada H, Yamada Y, Watanabe A, Tatsumi M, Yamashita J, Matsuda M, Sakaguchi T, Hirao T, Nakano H (1999). The prognostic significance of amplification and overexpression of c-met and c-erb B-2 in human gastric carcinomas. Cancer.

[14] Hara T, Ooi A, Kobayashi M, Mai M, Yanagihara K, Nakanishi I (1998). Amplification of c-myc, K-sam, and c-met in gastric cancers: detection by fluorescence in situ hybridization. Lab Invest.

[15] Tsugawa K, Yonemura Y, Hirono Y, Fushida S, Kaji M, Miwa K, Miyazaki I, Yamamoto H (1998). Amplification of the c-met, c-erbB-2 and epidermal growth factor receptor gene in human gastric cancers: correlation to clinical features. Oncology.

[16] Lennerz JK, Kwak EL, Ackerman A, Michael M, Fox SB, Bergethon K, Lauwers GY, Christensen JG, Wilner KD, Haber DA, Salgia R, Bang YJ, Clark JW, Solomon BJ, Iafrate AJ (2011). MET amplification identifies a small and aggressive subgroup of esophagogastric adenocarcinoma with evidence of responsiveness to crizotinib. J Clin Oncol.

[17] Kuniyasu H, Yasui W, Kitadai Y, Yokozaki H, Ito H, Tahara E (1992). Frequent amplification of the c-met gene in scirrhous type stomach cancer. Biochem Biophys Res Commun.

[18] Tsujimoto H, Sugihara H, Hagiwara A, Hattori T (1997). Amplification of growth factor receptor genes and DNA ploidy pattern in the progression of gastric cancer. Virchows Arch.

[19] Seruca R, Suijkerbuijk RF, Gartner F, Criado B, Veiga I, Olde-Weghuis D, David L, Castedo S, Sobrinho-Simoes M (1995). Increasing levels of MYC and MET co-amplification during tumor progression of a case of gastric cancer. Cancer Genet Cytogenet.

[20] Janjigian YY, Tang LH, Coit DG, Kelsen DP, Francone TD, Weiser MR, Jhanwar SC, Shah MA (2011). MET expression and amplification in patients with localized gastric cancer. Cancer Epidemiol Biomarkers Prev.

[21] Lee HE, Kim MA, Lee HS, Jung EJ, Yang HK, Lee BL, Bang YJ, Kim WH (2012). MET in gastric carcinomas: comparison between protein expression and gene copy number and impact on clinical outcome. Br J Cancer.

[22] Okamoto W, Okamoto I, Yoshida T, Okamoto K, Takezawa K, Hatashita E, Yamada Y, Kuwata K, Arao T, Yanagihara K, Fukuoka M, Nishio K, Nakagawa K (2010). Identification of c-Src as a potential therapeutic target for gastric cancer and of MET activation as a cause of resistance to c-Src inhibition. Mol Cancer Ther.

[23] Smolen GA, Sordella R, Muir B, Mohapatra G, Barmettler A, Archibald H, Kim WJ, Okimoto RA, Bell DW, Sgroi DC, Christensen JG, Settleman J, Haber DA (2006). Amplification of MET may identify a subset of cancers with extreme sensitivity to the selective tyrosine kinase inhibitor PHA-665752. Proc Natl Acad Sci U S A.

[24] Liu X, Newton RC, Scherle PA (2010). Developing c-MET pathway inhibitors for cancer therapy: progress and challenges. Trends Mol Med.

[25] Park WS, Oh RR, Kim YS, Park JY, Shin MS, Lee HK, Lee SH, Yoo NJ, Lee JY (2000). Absence of mutations in the kinase domain of the Met gene and frequent expression of Met and HGF/SF protein in primary gastric carcinomas. APMIS.

[26] Lee JH, Han SU, Cho H, Jennings B, Gerrard B, Dean M, Schmidt L, Zbar B, Vande Woude GF (2000). A novel germ line juxtamembrane Met mutation in human gastric cancer. Oncogene.

[27] Chen JD, Kearns S, Porter T, Richards FM, Maher ER, Teh BT (2001). MET mutation and familial gastric cancer. J Med Genet.

[28] Lee J, Seo JW, Jun HJ, Ki CS, Park SH, Park YS (2011). Impact of MET amplification on gastric cancer: possible roles as a novel prognostic marker and a potential therapeutic target. Oncol Rep.

[29] Graziano F, Galluccio N, Lorenzini P, Ruzzo A, Canestrari E, D'Emidio S (2011). Genetic activation of the MET pathway and prognosis of patients with high-risk, radically resected gastric cancer. J Clin Oncol.

[30] Albertson DG (2006). Gene amplification in cancer. Trends Genet.

[31] Vinatzer U, Dampier B, Streubel B, Pacher M, Seewald MJ, Stratowa C, Kaserer K, Schreiber M (2005). Expression of HER2 and the coamplified genes GRB7 and MLN64 in human breast cancer: quantitative real-time reverse transcription-PCR as a diagnostic alternative to immunohistochemistry and fluorescence in situ hybridization. Clin Cancer Res.

[32] Matsumoto K, Arao T, Hamaguchi T, Shimada Y, Kato K, Oda I, Taniguchi H, Koizumi F, Yanagihara K, Sasaki H, Nishio K, Yamada Y (2012). FGFR2 gene amplification and clinicopathological features in gastric cancer. Br J Cancer.

[33] Bachleitner-Hofmann T, Sun MY, Chen CT, Tang L, Song L, Zeng Z, Shah M, Christensen JG, Rosen N, Solit DB, Weiser MR (2008). HER kinase activation confers resistance to MET tyrosine kinase inhibition in MET oncogene-addicted gastric cancer cells. Mol Cancer Ther.

